# Understanding the effectiveness and mechanisms of a social prescribing service: a mixed method analysis

**DOI:** 10.1186/s12913-018-3437-7

**Published:** 2018-08-06

**Authors:** James Woodall, Joanne Trigwell, Ann-Marie Bunyan, Gary Raine, Victoria Eaton, Joanne Davis, Lucy Hancock, Mary Cunningham, Sue Wilkinson

**Affiliations:** 1 Leeds Beckett University, Leeds, LS1 3HE UK; 2 UK Coaching, Leeds, LS12 4HP UK; 3 University of York, York, YO10 5DD UK; 4 Leeds City Council, Leeds, LS2 8BB UK; 5 Leeds Mind, Leeds, LS18 4LB UK; 6 Connect for Health, Hillside, Beeston Road, Leeds, LS11 8ND UK; 7 NHS Leeds CCG’s Partnership, Leeds, LS16 6EB UK

**Keywords:** Social prescribing, Mixed-methods, Primary care

## Abstract

**Background:**

Evidence of the effectiveness of social prescribing is inconclusive causing commissioning challenges. This research focusses on a social prescribing scheme in Northern England which deploys ‘Wellbeing Coordinators’ who offer support to individuals, providing advice on local groups and services in their community. The research sought to understand the outcomes of the service and, in addition, the processes which supported delivery.

**Methods:**

Quantitative data was gathered from service users at the point they entered the service and also at the point they exited. Qualitative interviews were also undertaken with service users to gather further understanding of the service and any positive or negative outcomes achieved. In addition, a focus group discussion was also conducted with members of social prescribing staff to ascertain their perspectives of the service both from an operational and strategic perspective.

**Results:**

In total, 342 participants provided complete wellbeing data at baseline and post stage and 26 semi-structured qualitative interviews were carried out. Improvements in participants’ well-being, and perceived levels of health and social connectedness as well as reductions in anxiety was demonstrated. In many cases, the social prescribing service had enabled individuals to have a more positive and optimistic view of their life often through offering opportunities to engage in a range of hobbies and activities in the local community. The data on reductions in future access to primary care was inconclusive. Some evidence was found to show that men may have greater benefit from social prescribing than women. Some of the processes which increased the likelihood of success on the social prescribing scheme included the sustained and flexible relationship between the service user and the Wellbeing Coordinator and a strong and vibrant voluntary and community sector.

**Conclusions:**

Social prescribing has the potential to address the health and social needs of individuals and communities. This research has shown a range of positive outcomes as a result of service users engaging with the service. Social prescribing should be conceptualised as one way to support primary care and tackle unmet needs.

**Electronic supplementary material:**

The online version of this article (10.1186/s12913-018-3437-7) contains supplementary material, which is available to authorized users.

## Background

Social prescribing schemes remain in their relative infancy, but have become more popular in recent times [1]. Social prescribing has grown exponentially as a consequence of the growing voluntary and community sector’s role in health and intense pressure on primary care services to manage patients presenting conditions that can be addressed without medical intervention [2]. Such schemes provide General Practitioners (GPs) with a non-medical referral option that can be delivered alongside existing primary care services to improve individuals’ health and well-being [3]. Frequently patients are referred to befriending services, nature-based activities, volunteering opportunities, debt advice, bereavement groups or prescribed hobbies [1, 2, 4]. Social prescribing has been defined as:

“harness[ing] assets within the voluntary and community sectors to improve and encourage self-care and facilitate health-creating communities.” ([5], p.1)

Social prescribing is often delivered using link workers or social prescribers who are trained to act as the lynchpin between primary care services and organisations in the voluntary and community sector [2, 4]. The link workers’ ability to understand a social and holistic view of health is critical to the success of the schemes and moreover their interpersonal qualities are essential in the execution of social prescribing schemes and patients’ satisfaction of the service – a finding reiterated throughout the literature for over a decade [2, 5, 6].

The situation in primary care services – where service demand often outstrips supply – has been seen as a ‘wicked’ health problem and difficult to solve [7]. Despite the rhetoric that social prescribing offers a genuine way to reduce the burden on primary care services and moreover address unmet health and social needs of individuals and communities (see for example endorsement in the NHS Five Year Forward View [8] and the General Practice Forward View [9]), the effectiveness of such schemes are relatively unknown. Most social prescribing schemes lack evaluative components [10], with those that do showing very mixed results [5, 11]. More broadly, there is little evidence of the utility of partnerships between the voluntary and community sector and GP practices [7]. Commentators have argued, in fact, that the momentum and enthusiasm for social prescribing is unwarranted based on the evidence currently in existence [3]. This ‘evidence gap’ may be for a myriad of reasons, but mainly because the evaluation of schemes are often based on small-scale pilot studies that lack methodological rigour [3]. In addition, the aims of social prescribing schemes and their respective delivery models can have major variance [12] and so it is often challenging to synthesise, compare or pool data or information (although see Kimberlee’s proposed typology [13]). To date, there is no single model which encapsulates collaboration between GP practices and the voluntary and community sector [7].

A recent systematic review of social prescribing schemes emphasised the paucity of high-quality studies in this area, recommending more sophisticated designs to provide additional rigour [3]. Philosophically and ethically though, it seems that a randomised controlled trial to assess the effectiveness of social prescribing schemes is challenging and potentially morally and ethically contentious – in effect, this design would preclude patients accessing voluntary and community services to improve their health [14]. This point, however, is made more broadly about the evaluation of many health promotion and prevention-type services [15]. This had nevertheless not inhibited the attempts of researchers to undertake randomised controlled trials on social prescribing programmes in their early inception at the turn of the century [14], but such designs have not been replicated since [3]. Given that social prescribing services are usually relatively small-scale, research and evaluation budgets to determine effectiveness may not be sufficient to gather the ‘best’ evidence. The evidence base is, therefore, providing a very ‘mixed-picture’ which makes commissioning decisions on the implementation or continuation of social prescribing services troublesome [3].

The value of capturing both qualitative perspectives and quantitative outcomes of social prescribing services has been noted [16]. Using a mixed-methods design, this paper seeks to advance academic understanding of social prescribing and support future practice, policy and commissioning decisions. The paper’s focus is on a social prescribing service (referred to as ‘the service’) delivered in an area within a large city in Northern England. The paper presents data on the outcomes of the service in relation to health, well-being, social networks and GP utilisation and in addition the processes which contribute to these effects. It was not possible, due to funding and resource issues to adopt a control group to compare outcomes from those engaging with the service and those who did not. The service, in comparison to other social prescribing schemes reported in the literature [2], is relatively large with approximately 1500–2500 service users referred each year. To be eligible for the service, individuals must be 14 years and over and registered with a GP surgery. The service operates through ‘Wellbeing Coordinators’ who offer support to individuals and to provide advice on local groups and services in their local community – the activities individuals can be referred into range from mental health and counselling advice; physical fitness classes; support for physical or emotional difficulties; finance and debt advice; and creative groups. The Wellbeing Coordinator workforce is diverse in relation to age, ethnicity and professional experiences; however, all staff have a shared understanding of working in and with marginalised individuals and communities. Essential criteria on appointment to the role was the ability to be open, patient and flexible, non-judgemental and have the ability to empathise.

Individuals can self-refer into the social prescribing service or GP’s, health, social care and other relevant professionals can make a referral. Such diverse referral routes are characteristic of social prescribing schemes more generally [1]. Once referred, service user needs are either addressed directly over the phone (simple signposting for instance) or, in more intricate cases, they are provided with a one-to-one assessment to identify opportunities to explore their social support needs and provide them with information, support and guidance in relation to accessing local community activity to improve their health and wellbeing. Provision of the service is free at the point of access. After assessment, service users can potentially access a range of community and voluntary sector support. To avoid dependency on the social prescribing service, individuals are encouraged to ‘exit’ the service or are referred to other health and social care providers after 6 sessions. Most clients receiving appointments exit the service within 16 weeks, with the mean length of time being 10 weeks. As other social prescribing services have noted, the intervention is not suited to those with severe and complex need and indeed the limits on individuals receiving 6 sessions is to actively avoid individuals becoming dependent on the Wellbeing Coordinators [2].

## Methods

Qualitative methods have been identified as the most common way to assess social prescribing schemes, followed by the use of scales to measure well-being [9, 10]. This is perhaps unsurprising as the importance of capturing the ‘lived experience’ has been consistently emphasised alongside the measurement of outcomes using validated and reliable scales [12]. Fewer studies have combined both qualitative and quantitative approaches when understanding social prescribing services. This study, however, used a mixed-methods design to enable a holistic view of the social prescribing scheme. Approval for all aspects of the work was provided by Leeds Beckett University and NHS Yorkshire and Humberside Commissioning Support Unit.

In order to measure change in wellbeing, mental and physical health, social isolation and loneliness as well as ability to manage long term conditions, a questionnaire was administered by the Wellbeing Coordinators to clients at baseline (during the beginning of the initial assessment) and administered again when individuals ‘exited’ the service – this exit point could vary based on the individual and their circumstance but was usually within a six-week period. The questionnaire used validated measures, including: the Warwick-Edinburgh Mental Wellbeing Scale (WEMWBS); the EQ-5D – which covers five dimensions: mobility, self-care, usual activities, pain/discomfort and anxiety/depression; and the Campaign to End Loneliness Measurement Tool – a short 3-item scale – which examines social networks. The measures were chosen based on several factors which included reviewing existing literature to identify appropriate scales and practical considerations in relation to the length of time required to administer and complete the questionnaire. In addition, self-reported data on GP usage was also gathered. In some studies of social prescribing, ascertaining data has been problematic using quantitative tools with some reporting a very low response rate [17] – often due to quantitative tools not proving popular to administer by social prescribing workers. In this study, the Wellbeing Coordinators had input and commented upon the length, structure and format of the questionnaire which may have aided their acceptability in administering the tool. Data were input into SPSS with appropriate descriptive and inferential statistics conducted as appropriate. Care was taken, where appropriate, to disaggregate data by gender and age to provide a more nuanced understanding of the service outcomes.

From a qualitative perspective, interviews were conducted with social prescribing service users who completed pre- and post-questionnaire information and consented to be contacted by the research team to contribute further to the study. A total of twenty-six service users were interviewed by telephone over a twelve-month period in order to gather their views of the service. Commentators have asked for a more nuanced understanding of social prescribing schemes [12] – like what works for who and why? – and this was a key factor in our sampling strategy. Our purposive sampling approach [18], therefore, looked at important variables in the service user profile, which primarily included age, gender and the referral pathway experienced.

A semi-structured interview schedule was designed to explore broad topic areas, including: referral pathway, activities undertaken, perceived outcomes and benefits (or not) from the service, interaction with primary care services since engaging with the service and recommendations for service improvement (see Additional file 1). Individuals who took part in an interview were offered a high-street voucher to recognise their contribution. A focus group discussion was also conducted with seventeen members (15 Wellbeing Coordinators, the Service Manager and the Service Administrator) of social prescribing staff to ascertain their perspectives of the service both from an operational and strategic perspective. All interviews and the focus group discussion were recorded and transcribed after receiving written consent from all participants. Transcriptions were then analysed thematically by the research team to generate salient themes that emerged from the data. The analysis was informed by Braun and Clarke’s approach to thematic analysis [19]. Data from service users and staff were analysed separately, but several cross-cutting themes were identified in the analysis.

## Results

The results of the quantitative and qualitative data analysis have been synthesised in order to provide an overview of the outcomes of the service and to explore the processes that facilitate or inhibit the success of social prescribing schemes.

### Participant demographics

In relation to the pre- and post-questionnaire, 436 participants provided demographic information over an 18-month period. Of these, 63.9% of participants were female and 36.1% male – this was unrepresentative of the wider population where 50.9% are female. In total, 434 participants provided a date of birth which was used to calculate each person’s age at analysis. The mean age of participants was 53.1 years (SD = 18.02 years), with the oldest individual being 94 years old and the youngest 16 years. The largest proportion of individuals (21.4%) were aged between 50 and 59 years old – again this was not representative of the broader population where the largest proportion of individuals are aged between 20 and 24 years. Over half (56.5%) of participants were between 40 and 69 years old and 43.7% were under 50 years old. Table 1 shows that 86.9% of participants were White British this is broadly similar to the wider demography. Fourteen participants (3.2%) described their ethnic background as Asian/ Black Asian.

**Table 1:** Ethnicity of participants

	Frequency	Percent
White British	379	86.9%
White Irish	10	2.3%
Other White	6	1.4%
Mixed White and Black Caribbean	3	0.7%
Mixed White and Black African	2	0.5%
White and Asian	2	0.5%
Asian and Black Asian	14	3.2%
Black Caribbean	3	0.7%
Black African	8	1.8%
Other Black African	2	0.5%
Other	7	1.6%

Of the 26 service users who participated in a semi-structured interview, 14 were male and 12 were female. The sample had accessed the service through a range of referral routes and had experienced a range of different voluntary and community support activities.

### Wellbeing

In total, 342 participants provided complete wellbeing data at baseline and post stage over an 18-month period. During the same 18-month period approximately 2250–3750 service users were in contact with the service – this contact, however, could vary between a single telephone enquiry, to individuals accessing the maximum of 6 sessions with a Wellbeing Coordinator. In many cases, the vast majority of participants were lost to follow-up or did not engage with the service after the initial assessment period and therefore did not complete post-measures.

Of the 342 participants providing data at baseline and post stage, 265 (77.5%) had an improved wellbeing score from baseline to post stage; 58 (17%) had a decrease in score; 19 (5.6%) had no overall change. For the cohort, the average wellbeing score at baseline was 18.16 (SD = 6.03) while at the post stage the average score was 22.13 (SD = 5.8). The average change in score was 3.98 (SD = 5.33) with a 95% confidence interval of 3.41 to 4.55. The results of a paired t-test suggested there was a statistically significant improvement in well-being from baseline to post stage (*t* = 13.81, df = 341, *p* < 0.001). The size of the improvement was medium to large (*d* = 0.75).

As demonstrated by Table 2, the average wellbeing score improved significantly from baseline to post stage for both males and females. There was no significant difference between males and females in terms of improvement in wellbeing (*t* = 0.47, df = 337, *p* = 0.96).

**Table 2:** Wellbeing score by sex

	Mean Baseline (SD)	Mean Post (SD)	Mean Change (SD)	95% CI	T (df)	Sig
Males (*n* = 127)	18.19 (5.76)	22.14 (5.86)	3.95 (5.78)	2.94 to 4.97	7.71 (126)	*p* < 0.001
Females (*n* = 212)	18.15 (6.21)	22.13 (5.82)	3.98 (5.08)	3.29 to 4.67	11.4 (211)	*p* < 0.001

Analysis revealed there to be a significant negative relationship (*r* = − 0.21, *p* < 0.001) between age and change in wellbeing from baseline to the post stage. This indicates that younger individuals engaging with the service tended to have greater improvement in wellbeing than older people. To explore the relationship between age and wellbeing, individuals were assigned into 2 groups i) under 50 years, ii) 50 years and older. An analysis of change in wellbeing scores over time was then conducted. Table 3 shows that the average wellbeing score improved significantly from baseline to post stage for both age groups. Additional analysis revealed that average improvement was significantly greater in the under 50 years age group (4.74) than the 50 years and over group (3.42) (*t* = 2.43, df = 336 *p* = 0.02).

**Table 3:** Wellbeing score by age group

	Mean Baseline (SD)	Mean Post (SD)	Mean Change (SD)	95% CI	T (df)	Sig
Under 50 (*n* = 139)	17.56 (5.7)	22.3 (5.71)	4.74 (4.94)	3.91–5.57	11.32 (138)	*p* < 0.001
50 & over (*n* = 199)	18.58 (6.23)	22.01 (5.91)	3.42 (5.57)	2.64–4.2	8.67 (198)	*p* < 0.001

Improvements in wellbeing were reflected in the qualitative interviews with individuals describing feelings of optimism and a more positive outlook as a result of being referred to the service. Other individuals reflected on how social prescribing had impacted positively on their wellbeing through offering occupational engagement. Accessing a range of activities such as swimming and ‘hobby’ activities provided a greater sense of independence and gave individuals a sense of purpose:

*“I’d been feeling very depressed, I’ve been in the building trade for fifty years very active, doing all my own repairs at home I was a joiner. And then I’m suddenly stuck in a wheelchair. And it was more frustration. In my mind I could still do the job but physically I couldn’t. And everything was load onto my wife. You know she was having to do things that I used to do I had to sit and watch her …and it just got me down. Still does at times… the service just gave me suggestions on things to do like one thing I’ve always enjoyed is swimming. And I haven’t done it for years. And it was you know accessing things like that. There is a workshop where people go to do wood work…I feel a bit better in myself knowing that there are things out there that I can do.” (Male client: interview 12, aged 50 years and over. Referred to the social prescribing service by GP)*

Having the opportunity to attend support groups in the local community, facilitated through the social prescribing service, enabled individuals to gain more of a balanced perspective by being able to share experiences with others going through similar difficulties. This resulted in some individuals feeling much more hopeful about their own lives:

*“Since I went to that group I could see what other people are actually having difficulty in life with, and you do not assess yourself the same. It actually made me realise that life is not all about yourself. You find here that everybody has got different problems. You find that yours is not even as serious as the other person that you are talking to.” (Female client: interview 2, aged under 50 years. Self-referred to the social prescribing service)*

### Health and functioning

**Fig. 1:**
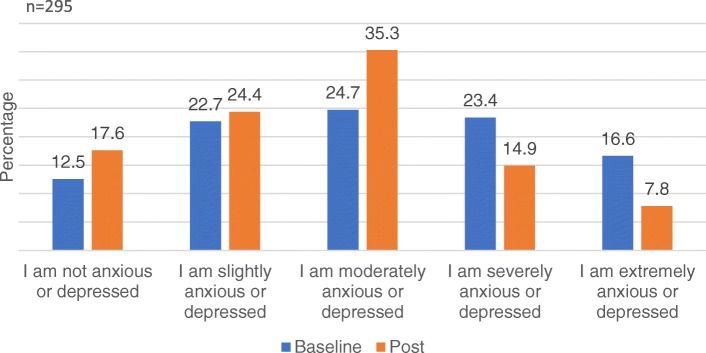
Level of anxiety/depression at baseline and post stage

The questionnaire measured the extent to which respondents rated their health and functioning. In addition, participants also indicated how anxious or depressed they felt today. Fig. 1 shows sizeable decreases from baseline to post stage in the proportion of participants who reported being severely or extremely anxious or depressed (*n* = 295). At baseline, 40% reported being either ‘severely’ (23.4%) or ‘extremely’ anxious or depressed (16.6%). At post stage, 22.7% were ‘severely’ (14.9%) or ‘extremely’ anxious or depressed (7.8%). Notably, the proportion of individuals feeling ‘moderately’ anxious or depressed increased from 24.7 to 35.3% from baseline to post stage. Results also showed an increase in the proportion of individuals who were not feeling anxious or depressed across time (12.5% to 17.6, baseline to post stage). Levels of anxiety or depression at post stage were significantly lower than at baseline (z = − 5.47, *p* < 0.001).

Participants were asked to rate their health today on a scale of 0 to 100, where 0 was the ‘worst health you can image’ and 100 was the ‘best health you can imagine’. Out of the 320 participants: 191 (59.7%) had an improved health rating score from baseline to post stage; 76 (23.8%) had a decrease in score; and 53 (16.6%) had no overall change. Analysis showed a statistically significant improvement in health from baseline to post stage (*t* = 7.64, df = 319, *p* < 0.001) (95% CI: 7.09 to 12.02). The average health rating at baseline was 43.27 (SD = 20.87) compared to 52.83 (SD = 20.83) at the post stage. The size of the improvement in health rating was small to medium (*d* = 0.43).

As can be seen from Table 4, average health rating improved significantly from baseline to post stage for both males and females. The increase amongst males was notably higher than in females, but the analysis suggested that the difference was not statistically significant *t* = 1.34, df = 315, *p* = 0.18) (95% CI: -1.63 to 8.6).

**Table 4:** Health rating by sex

	Mean Baseline (SD)	Mean Post (SD)	Mean Change (SD)	95% CI	T (df)	Sig
Males (*n* = 113)	43.5 (19.34)	54.96 (19.47)	11.46 (21.31)	7.49–15.43	5.72 (112)	*p* < 0.001
Females (*n* = 204)	43.51 (21.57)	51.5 (21.51)	7.98 (22.63)	4.86–11.11	5.04 (203)	*p* < 0.001

Table 5 shows that average health rating improved significantly from baseline to post stage for both age groupings (under 50 year old and 50 and over). There was found to be no significant difference in average health rating change over time between the 2 groups (*t* = − 0.26, df = 314, *p* = 0.79) (95% CI: -5.79 to 4.43).

**Table 5:** Health rating by age group

	Mean Baseline (SD)	Mean Post (SD)	Mean Change (SD)	95% CI	T (df)	Sig
Under 50 (*n* = 120)	44.13 (20.48)	53.15 (22.09)	9.02 (21.53)	5.12–12.91	4.59 (119)	*P* < 0.001
50 & over (*n* = 196)	42.91 (21.16)	52.61 (20.25)	9.7 (22.92)	6.47–12.93	5.92 (195)	*p* < 0.001

### Social networks

The average ‘social networks’ score at baseline was 9.09 (SD = 2.68) and at the post stage, the average score was 9.92 (SD = 2.37). The average change in score was 0.83 (SD = 2.35) with a 95% confidence interval of 0.57 to 1.1 which indicates significant improvement in relationships and social networks. A paired t-test also revealed a statistically significant improvement in ‘Social networks’ from baseline to post stage (*t* = 6.21, df = 305, *p* < 0.001). The size of the improvement was small/medium (*d* = 0.35). Out of the 306 participants, 155 (50.7%) had an improved ‘Social Networks’ score from baseline to post stage; 76 (24.8%) had a decrease in score; and 75 (24.5%) had no overall change.

There was statistically significant improvement from baseline to post stage for both age groupings (under 50 years and 50 years and over, see Table 6). Average improvement in ‘Social networks’ score was significantly greater in the under 50 years old group than the 50 years and over group (*t* = 2.13, df = 302, *p* = 0.03).

**Table 6:** ‘Social networks’ score by age

	Mean Baseline (SD)	Mean Post (SD)	Mean Change (SD)	95% CI	T (df)	Sig
Under 50 (n = 120)	8.68 (2.5)	9.86 (2.43)	1.18 (2.43)	0.75 to 1.62	5.35 (119)	*P* < 0.001
50 & over (*n* = 184)	9.33 (2.77)	9.93 (2.33)	0.6 (2.35)	0.27–0.93	3.55 (183)	*P* < 0.001

One of the salient themes to emerge from the qualitative analysis was the improved sense of social connectedness as a result of engaging with the social prescribing service. Many of the respondents reported feeling socially isolated prior to engagement with the service:

*“I felt isolated, because my husband works away and the kids are at school. I felt very isolated and I knew that wasn’t going to do me any good, so it was a case of I wanted to get into the community and meet people and things. So it was a case of oh well we’ll see what groups there are and see how we can link it to your hobbies.” (Female client: interview 6, aged under 50 years. Self-referred to the social prescribing service)*

*“I used work to hide. And it’s when you stop you think great I’ve got all this time. But after a while…you can be married but you can be lonely. Because she (wife) works funny hours, I lost that day time interaction with people, and I think that’s the problem.” (Male client: interview 7, aged under 50 years. Referred to the social prescribing service by Primary Care Nurse)*

Interviewees suggested that the Wellbeing Coordinators offered a myriad of opportunities to engage in social activities to alleviate isolation. The range of services on offer meant that, in many cases, there was compatibility between the interests of service users and the activities available:

*“She got me involved in a walking group. She found me another number for a dancing group. She did really well for me to be honest. She was what I was looking for at the time, to get myself out of the property and do things.” (Male client: interview 10, aged 50 years and over. Self-referred to the social prescribing service)*

The Wellbeing Coordinators suggested that they had worked consistently to build a presence in various geographical areas, building relationships with a number of different services and organisations and understanding the local offer in communities and neighbourhoods:

*“Individually and collectively they [Wellbeing Coordinators] have worked really hard to get foot hold in their areas, becoming part of forums, neighbourhood networks, health and wellbeing partnerships… and they’re not easy to get into, particularly because it’s quite a difficult structure to understand, the health and the local area officer patches. It’s quite complicated and the team have worked really hard to become involved in those things.” (Wellbeing Coordinator Manager)*

Several service users described a link between their increased feelings of social connectedness and their overall sense of confidence and purpose. In some cases, this had tangible effects on close relationships and had strengthen interpersonal connections:

*“I have two teenage daughters. I think it was very tough for them. They didn’t understand other than the fact they could see mum was really upset and struggling with things. And they did help a great deal and they are still helping me although not as much as they did [Laughs].” (Female client: interview 9, aged under 50 years. Referred to the social prescribing service by GP)*

*“My husband says he can notice a difference when I’ve been out and done something rather than staying at home all day. It’s given us more to talk about as well.” (Female client: interview 10, aged 50 years and over. Self-referred to the social prescribing service)*

### Use of GP services

At baseline, participants were asked about their current GP usage and around half of participants (48.6%) reported going to the GP less than once every month and a further 30.6% visited every 3 or 4 weeks. Fifteen percent (15.1%) went every 2 weeks and 5.7% visited at least once a week. At post stage, individuals were asked about their use of GP services since participating in the social prescribing service. A majority of participants (53.3%) reported using GP services about the same. Notably, 27.2% of participants had used services less, with 5.5% visiting ‘a lot less’ and 21.7% a ‘bit less’. Conversely, 19.4% reported increased GP use, with 4.9% visiting ‘a lot more’ and 14.5% a ‘bit more’.

**Fig. 2:**
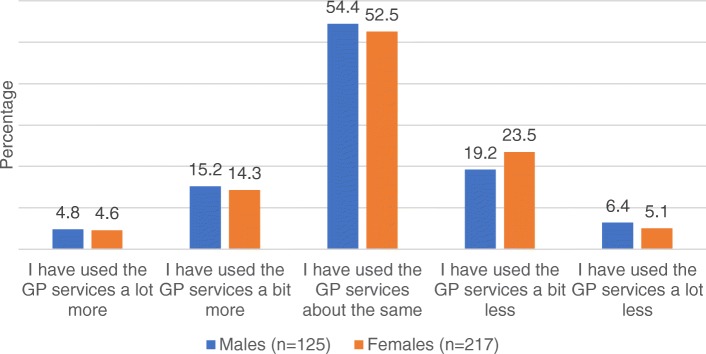
GP usage at post stage by sex

Figure 2 provides a breakdown of GP usage at post stage by sex. Analysis revealed there to be no significant sex difference in the use of GP services at the post stage (U = 13,202.5, *p* = 0.66).

**Fig. 3:**
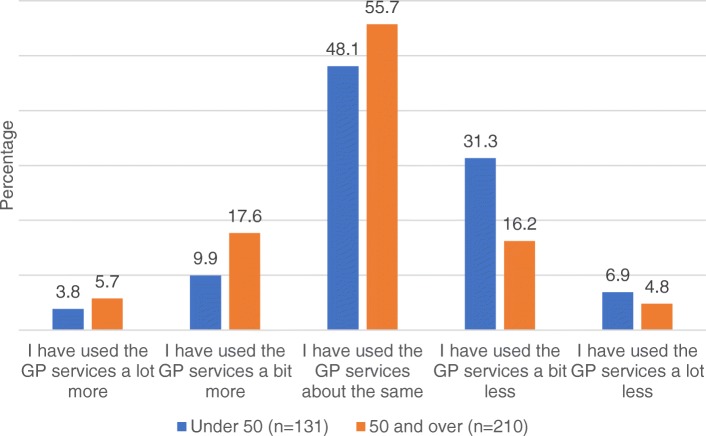
GP usage at post stage by age

Figure 3 provides a breakdown of GP usage at post stage by age. Analysis revealed there was a significant age difference in the use of GP services at the post stage (U = 10,963, z = − 3.44, *p* = 0.001), with participants 50 years and over using the GP services more at post stage compared to the under 50’s.

Similar to the quantitative results, the qualitative interviews resulted in mixed responses in relation to GP usage. For example, some individuals stated that their contact with the GP had reduced since their involvement with the social prescribing service due to a more positive state of mind:

*“I cut down by seeing them because obviously well basically because I seemed better in myself. You know, and more happy because you know the progress that I’ve made.” (Male client: interview 5, aged under 50 years. Referred to the social prescribing service by a voluntary-sector provider)*

However, other individuals stated that their GP use had not changed and for some their usage had increased due to having a greater awareness of their own health needs after engaging with the social prescribing service.

### Processes facilitating the success of social prescribing

The qualitative data highlighted a number of factors that were critical to the success of the social prescribing service. These are presented here.

#### The attributes of the Wellbeing Coordinator

Qualitative analysis demonstrated the critical role of the Wellbeing Coordinator in facilitating successful outcomes for individuals. Service users described how the informal and yet informative nature of the interactions with the Wellbeing Coordinators had enabled them to better consider their own health needs. The interpersonal qualities of the Wellbeing Coordinator were raised as a key factor in service users engaging with the social prescribing service. The approachability, trustworthiness and communication skills of the Coordinator were crucial and often resulted in individuals feeling valued and listened to:

*“And to be taken notice of. And to be looked on as a person as an individual as opposed to ‘oh just somebody else’.” (Female client: interview 9, aged under 50 years. Referred to the social prescribing service by GP)*

*“It’s that being able to talk to somebody, and somebody being willing to listen, I think that’s the crux of it, and not being judgmental.” (Male client: interview 12, aged under 50 years. Referred to the social prescribing service by GP)*

Both service users and staff discussed the co-constructed and consultative nature of the service. Rather than being dictated to, clients felt assured that the process was very much about working together to decide on the best course of action:

*“I was free at any time to say ‘no I’m not comfortable with this I don’t like it’ and she was very adamant that it would not affect me if she’d arranged it all and I’d have gone and then come back and said ‘no I can’t do this’ she’d have been fine with that. It was kind of all along how I felt and she made that very clear that any time that I didn’t feel comfortable with anything that she maybe suggested or got me to have a look at, if I didn’t like the idea it was no problem.” (Female client: interview 3, aged 50 years and over. Referred to the social prescribing service by GP)*

*“It’s very much an enabling process to enable the person to address the issues that are relevant, the non-medical issues…and so to fit all that together and come up with an action plan.” (Wellbeing Coordinator)*

#### Engaging men

Extending the importance of interpersonal relationships between service users and the Wellbeing Coordinators was the evidence which suggested that the service had been particularly helpful to male clients in allowing them to express their emotions without having to live up to a gender stereotype:

*“At first when she (worker) mentioned mental health I thought I’m not one of those, but when I look back it’s a massive problem...mental health issues for men are massive. I’m not saying women don’t have the same problems, but if you see a woman crying you don’t think oh soft sod.” (Male client: interview 1, aged 50 years and over. Referred to the social prescribing service by GP)*

For some of the men interviewed, this feeling of being able to speak more openly about particular issues seemed to stem from the fact that their interactions were with a female Wellbeing Coordinator. Some interviewees reported that they were better able to relate to women and perceived that female staff could offer more compassion and empathy:

*“I just seem to have a better response talking to females to be honest. I can’t put my finger on it as to why that is. It just seems to be a little calmer in coming back to me and things like that…I do get on with males as well but a female seems to be more helpful.” (Male client: interview 13, aged under 50 years. Self-referred to the social prescribing service)*

One client suggested that had it been a male worker he was consulting with, he may not have been as open to discussions:

*“I would have been a bit more wary as to how open I would have been with a male.” (Male client: interview 5, aged under 50 years. Referred to the social prescribing service by a voluntary-sector provider)*

#### Flexibility and duration of the service

Some respondents suggested that the flexibility of the service had been a strength, enabling individuals to have control over when and how they accessed the Wellbeing Coordinators and/or activities in the community:

*“She told me that she was only allowed to see me for six sessions or something like that, so I decided that I wanted it every fortnight, to three weeks maybe, just to make it last a bit longer. Cos she was helping. And then sometimes when I just wasn’t feeling it I’d cancel it and meet up the next week.” (Male client: interview 13, aged under 50 years. Self-referred to the social prescribing service)*

This particular service offered service users six sessions with a Wellbeing Coordinator to plan and facilitate client-centred activities. A number of interviewees, however, stated that it would be useful to have a greater number of one to one sessions should they need to:

*“I think it probably could have been longer. I think it should be more like help until they think they are done. Cos when I first met her I was really down, but towards the end I was much better but I still could have done with one or two more.” (Male client: interview 4, aged under 50 years. Referred to the social prescribing service by GP)*

*“The time wasn’t really enough. I wish the time was a bit extended. I requested more time. It’s not enough time to sort out everything that a person would actually want to do. What I wanted we couldn’t really sort out everything.” (Female client: interview 2, aged under 50 years. Self-referred to the social prescribing service)*

#### Understanding the voluntary and community sector

The success of the social prescribing service was heavily contingent on the resources available in the voluntary and community sector in the area. Without a range of options to offer to service users, the social prescribing service may not be able to address the needs of all individuals. To allow understanding of the voluntary and community sector, Wellbeing Coordinators discussed how they maintained a good working knowledge of the assets in the community through engaging directly with organisations or through ‘umbrella’ groups representing the voluntary and community sector:

*“We’ve had the time to go out and research our areas, and we’ve met managers or teams who think their services will be beneficial to our clients. And we’ve arranged to go to team meetings, explain our service to them so they are aware of us so they can refer to us and we can also refer to them.” (Wellbeing Coordinator)*

Moreover, some Wellbeing Coordinators had been given specific responsibility to focus on the resources and activities available to certain sub-groups of the population in order to ensure that the needs of all service users could be addressed:

*“What we are doing now is we are putting champions in areas, so people are taking leads on…older people, mental health so we’ve got specific areas that people can learn more about and then if one of our Wellbeing Coordinator has someone they don’t know where to send, they can go to that champion and they might have a better idea of where to turn to.” (Wellbeing Coordinator)*

## Discussion

There has been a growth in the commissioning and delivery of social prescribing services in communities across the UK. This attempt to divert patients from primary care services and GP practices to the voluntary and community sector is laudable, but evidence of effectiveness to support such decision-making has been lacking. The reasons for this evidence gap are manifold and may link to social prescribing services often delivered as short-term pilots with small, or no, evaluative component [3]. This paper sought to assess the outcomes of one social prescribing service in Northern England, using both qualitative and quantitative methods. Such mixed-method approaches have not been common in the published literature [3].

This research provides promising evidence of outcomes and highlights key process issues for social prescribing services. This research is unique in providing insight into the outcomes of the service based on gender and age. This is relatively uncommon in the published literature. The research sought to provide both a viewpoint of the service from the perspective of those directly involved in delivering and those receiving support from the service. Alongside this, service-user questionnaire data was gathered pre and post contact with the social prescribing service. The research shows very positive and promising outcomes for social prescribing and moreover demonstrates the potential to address individuals’ needs in a holistic way. Improvements in participants’ well-being and perceived levels of health and social connectedness as well as reductions in anxiety has been shown. In many cases, the service had enabled individuals to have a more positive and optimistic view of their life often through offering opportunities to engage in a range of hobbies and activities. Finding these occupational engagement and enrichment activities is often an important element in efforts to promote health with those referred to social prescribing schemes [2, 5, 20]. The findings here provide a platform to allow other research to examine further the impact of social prescribing schemes on different populations groups. This potentially enables a more nuanced view of social prescribing, rather than current perceptions that social prescribing is a universal intervention to benefit all. This research, for example, has provided some evidence to show that men may have greater benefit from social prescribing than women. Such claims, however, would require further validation.

The role of the Wellbeing Coordinator seems to be a critical ingredient of success. Spending time getting to know their service users and working from a more flexible approach enabled the Wellbeing Coordinators to develop trusting relationships, which ultimately seems to be a factor for the on-going engagement of communities. Individuals appeared to appreciate the consistency of the service as well as follow ups to ensure their progress. Previous studies have also shown how important the social prescriber’s role is in service users achieving optimal gains from social prescribing services [5]. This finding has particular implications for the recruitment and training of those working in social prescribing roles, given that their role is critical. Research, for example, has shown that staff attrition can be a major hindrance in delivering effective social prescribing services [1].

Many social prescribing services have sought to assess the impact of their schemes on reduced healthcare utilisation, particularly GP and primary care services. This exploration has been very attractive for commissioners, with some social prescribing services reporting that service users are more likely to find community-based solutions to health and social issues rather than accessing health systems [21]. While understanding the impact is useful, especially for those looking to social prescribing to provide the ‘magic bullet’ to minimising the burden on primary care services ([2], p.317), a note of caution should be exercised. Social prescribing schemes may heighten individuals’ awareness of their own health and this may uncover further needs that may require primary care intervention [12]. This was evidenced in this study where participants articulated some reluctance to use GP services less, but this was by no means a universal position. Indeed, some participants suggested that their usage had been unchanged but that the service had enabled a focus on aspects of their health that were not previously managed by their GP. Other recent studies have drawn similar conclusions [16] which provides somewhat of a dilemma and is perhaps why reductions in primary care utilisation is not a useful marker for the effectiveness of social prescribing [11]. Our research focused particularly on GP usage, but future research may consider a more nuanced strategy and explore the impact on other healthcare professions.

Social prescribing services are often hinged on effective relationships between voluntary and community sector providers and primary care services [18]. Research indicates that these relationships can be precarious and fragile [7] and require constant attention and nurturing. Indeed, the strength of the voluntary and community sector is critical to the development of social prescribing interventions [2] – this study, based in a large city in Northern England, has a strong third sector infrastructure which enabled service users to be supported. The success, in part, was arguably contingent on the local activities that people can be ‘referred-out’ to [22]. This finding raises broader concerns about the sustainability of third sector providers in delivering health and social care activities and the potential dangers in overburdening small-scale organisations through social prescribing diverting individuals away from primary care services [2]. This is particularly important given the impact that austerity has had on the voluntary and community sector [23]. Indeed, for this intervention to work, social prescribing services require a strong and vibrant voluntary and community sector which requires appropriate funding and infrastructure for success.

The key strengths of this study lie in the mixed-method design, something that is not commonly found in the current evidence base [16]. The integration of quantitative and measurable changes in service-user outcomes and rich, in-depth qualitative narratives allow greater understanding and nuance in our understanding of social prescribing services. This study has limitations in design, as it was not possible to have a control group and its focus on only one social prescribing service. On the latter point, the diversity of social prescribing models and practice is clear but key principles are shared across delivery mechanisms [10] which can enable transferability and generalisability of findings. In relation to the former point, it was not possible to randomise service-users to a control or intervention group or make comparisons with service-users not referred to social prescribing services. The ethical and practical challenges of such designs in this context have been noted previously [15]. Also, those individuals providing complete quantitative data at baseline and post stage is a relatively small proportion in comparison to the total number of service users engaging with the social prescribing service. This study only accessed those completing the allotted 6 sessions and who provided data at both time-points. In addition, there were limitations in relation to the qualitative aspects of the study as interviews with participants and the focus group with staff were cross-sectional in design. During the focus group with social prescribing staff, there was potential for views to have been withheld by the Wellbeing Coordinators as the service manager was present during the discussion. There was no evidence to suggest that this was the case, however.

## Conclusion

This study has shown promising findings in relation to the value and the positive outcomes that can arise through a social prescribing service. Moreover, the study has identified key processes that make successful outcomes from social prescribing more likely. Criticisms that social prescribing services are simply the latest ‘shiny new policy thing’ ([22], p.90) seem now to be unjustified given the evidence and support for such approaches since the start of this century [2]. However, it is very clear that the research evidence on social prescribing has not kept pace with policy direction and momentum. As an example, the extension and enthusiasm for social prescribing into new domains such as dementia may be creditable, but it has shown limited effects in the research literature [24]. It is anticipated that this research, and other studies focussing on social prescribing, will encourage commissioners to conceptualise social prescribing as part of a longer-term re-orientation of primary care services [25] where medical and social models of health come together to provide a more positive experience for individuals and communities.

## Additional file

Additional file 1: Interview Schedules. (DOCX 14 kb)

## Abbreviations

GPs: General Practitioners
WEMWBS: Warwick-Edinburgh Mental Wellbeing Scale
