# Supplementary material for: Understanding the effectiveness and mechanisms of a social prescribing service: a mixed method analysis
Source: BMC Health Serv Res. 2018 Aug 6;18:604. doi: 10.1186/s12913-018-3437-7 (PMC6080378; doi:10.1186/s12913-018-3437-7)
Supplement: Supplementary file 1 — Interview Schedules. (DOCX 14 kb) [file 12913_2018_3437_MOESM1_ESM.docx]

Interview Schedule (Service Users)

1. Can you tell me something about the nature and history of your involvement with the Social Prescribing Service?
2. What services, if any, were you accessing prior to the Social Prescribing Service?
3. How did you hear or become aware of the service?
4. What were your expectations of the service and how it would support you?
5. What was your experience of your initial assessment with the Community Advisor?
6. Can you explain what happened after the initial assessment? Were you able to link in with a service to support your needs?
7. Did you have regular contact with the Community Advisor? If so, what was discussed? Was this useful?
8. What difference, if any, has the Social Prescribing Service made to you?
   1. Probe impacts on the wider family too.
9. Has your contact with the GP practice changed in any way since being involved in the Social Prescribing Service?
10. Are there any ways in which the service could be improved or modified to better support people?
11. Do you have any recommendations for the service?

Interview Schedule (Staff)

1. Can you tell me about your role with the Social Prescribing Service?
2. Could you describe how the service operates from your perspective?
   1. Referral system and referral criteria
   2. Support offered to service users
   3. How service users ‘exit’ the service
3. From your experiences, in what ways does the service support service users?
   1. What difference do you see the service making to people?
   2. What role, if any, do you think the service ‘diverts’ people from primary care?
4. What have been the main challenges in delivering the service?
   1. To what extent has the voluntary and community sector been able to provide services for service users?
   2. In what ways has communication and partnerships between the Social Prescribing Service and the voluntary and community sector been established?
   3. In what ways has communication and partnerships between the Social Prescribing Service and GP/primary care sector been established?
5. Are there any ways in which the service could be improved or modified to better support people?
6. What do you see are the key strengths of the social prescribing model?
7. Do you have any recommendations for the service?
